# Emotional, Cognitive, and Social Factors Influencing Romanian Women’s Intention to Undergo Cervical Cancer Screening: A Mixed-Method Study

**DOI:** 10.3390/healthcare13172147

**Published:** 2025-08-28

**Authors:** Nicoleta-Monica Pașca, Diana Taut, Sebastian Pintea, Adriana-Smaranda Băban

**Affiliations:** Department of Psychology, Babeș-Bolyai University, 400347 Cluj-Napoca, Romania

**Keywords:** cervical cancer screening, cervical cancer, HPV infection, intention

## Abstract

**Objective:** To identify emotional, cognitive, and social factors associated with Romanian women’s intention to undergo cervical cancer screening (CCS). **Methods:** An online, cross-sectional, mixed-method survey was conducted among Romanian women. Quantitative statistics were performed to uncover associations, while inductive content thematic analysis was used to refine results. **Results:** 317 women responded. Previous history of CCS (OR = 7.564, CI = 3.523–16.241, *p* < 0.001), testing positive for HPV strains (OR = 30.176, CI = 1.828–498.049, *p* < 0.001), knowing that the infection can cause CC (OR = 4.398, CI = 1.117–14.994, *p* < 0.017), believing the infection is asymptomatic (OR = 2.919, CI = 0.883–8.411, *p* = 0.04 and being aware of the HPV vaccine (OR = 5.56, CI = 1.722–16.649, *p* = 0.002 were associated with the intention to undergo screening. Fear of receiving a cancer diagnosis (OR = 2.727, CI = 1.291–5.764, *p* = 0.009) was associated with higher intention to undergo screening, while shame and feelings of violated privacy negatively impacted the intention. High perceived chances of being infected (OR = 3.38, *p* = 0.002) and perceiving CCC as beneficial (OR = 7.634, *p* < 0.001) drove respondents to pursue CCS. Doctor’s recommendation (OR = 15.357) and partner’s support were associated with the intention to pursue CCS when anticipating an HPV infection (OR = 6.016, CI = 2.808–12.888, *p* < 0.001) or a diagnosis of CC (OR = 4.794, CI = 2.267–10.139, *p* < 0.001). When anticipating a diagnosis of CC, guilt (OR = 2.557) and fear of dying (OR = 2.253) were significant factors impacting women’s intention to screen. Qualitative analyses uncovered limited knowledge regarding HPV, a lack of awareness about the CCS’s advantages, low perceived susceptibility, and financial burden as factors deterring women from screening. Fear, responsibility, and previous personal or family history of cancer or symptoms were the main drivers motivating participants to engage in CCS. **Conclusions:** Findings highlighted key emotional, cognitive, and social factors that influence Romanian women’s intention to undergo CCS. This analysis can serve as a foundational support for developing future tailored interventions designed to address low addressability within the targeted population, especially as Romania is in a nascent state of creating population-based screening programs.

## 1. Introduction

Cervical cancer (CC) is the 4th most common neoplasia in women worldwide, with an incidence of 662,044 new cases and 348,709 deaths reported in 2022 [1]. Despite readily available primary and secondary preventive measures, such as the Human Papillomavirus (HPV) vaccine and screening for precancerous lesions, CC remains one of the most challenging preventable oncological pathologies [2], especially in low and middle-income countries [3].

In Europe, there are significant discrepancies in the incidence and prevalence of CC between countries. Romania has the highest European incidence, with a soaring Age-Standardized Rate (ASR) of 27.1 per 100,000. In contrast, Switzerland has the lowest incidence, with ASR as low as 4.1/100,000 [4]. These differences can be accounted for by different policies surrounding primary and secondary prophylaxis. According to the HPV Prevention Policy Atlas of 2023, these different policies regarding cervical cancer screening (CCS) pertain to screening availability, type of screening test performed (HPV typization, cervical cytology, or both), availability of at-home testing (i.e., self-sampling), and reimbursement of costs [5].

In Romania, the national CCS program lacks stable funding from the central budget and instead relies heavily on intermittent external European sources. There is no centralized registry or fully organized national system in place, only fragmented regional initiatives, while opportunistic screening remains the dominant approach [5]. As a result, Pap smear testing is not consistently available, with services being offered irregularly and without predictability. As a consequence, CCS coverage was as low as 39% in women aged 35 to 49 [6].

In addition to structural barriers, such as the lack of appropriate data infrastructure or systematic invitations for targeted populations and the absence of clear pathways and well-established policies [7,8], several individual barriers contribute to screening addressability. Socio-demographic factors, such as age, education level, ethnicity, and income, seem to impact the addressability and access to screening [9,10,11]. For instance, low socio-economic conditions were consistently associated with high susceptibility to the disease and low perceived benefits of attendance [12]. Cognitive factors such as insufficient awareness or knowledge have been identified as pivotal elements that can deter women from adhering to screening practices [13,14,15]. In psychology, several cognitive theories have strived to explain health-related behaviours, including the uptake of screening practices such as CCS. For example, the Health Belief Model (HBM) was previously deployed to explain adherence to CCS [16], and some constructs comprising the theoretical model, such as perceived benefits of CCS and disease severity, have been correlated with the intention to screen [17,18].

Within the border of social and cognitive constructs, the Theory of Planned Behaviour (TPB) is another model that studies human intention and behaviour [19]. Constructs influencing intention, like perceived behavioural control, perceived social pressure, and personal attitude, were all associated with screening intention [20,21,22]. These previous findings, encompassing cognitive theories and their importance in explaining prophylactic behaviours, showcase the complex interplay between cognitive assessment, emotions, and behaviours. As such, emotions have been proven to play a crucial role in health-related behaviours. Few studies have managed to explore emotional components, such as anticipated emotions or anticipated regret, through the TPB model, linking anticipative emotions to screening uptake, including CCS [23,24,25,26,27].

The concept of anticipated emotion refers to an emotion that one is expected to experience when faced with a future event [28]. Nevertheless, anticipating an emotional reaction requires a cognitive assessment. Thus, various publications have explored anticipated emotions in this context. According to Lazarus’s Appraisal Theory, emotion results from an individual’s appraisal of goal congruence or incongruence, expectation, and sense of control [29]. Emotional reactions such as fear, anxiety, embarrassment, or the feeling of privacy violations sway women away from screening [17,30,31]. However, these barriers carry different weights in different regions and across diverse socio-economic contexts.

Vulnerability is a key factor influencing CCS attendance, with recent research increasingly focusing on hard-to-reach or underserved populations [12,32]. In Romania, however, CCS participation remains alarmingly low despite public health recommendations, while the incidence of CC is among the highest in Europe. This challenge is further exacerbated by the absence of comprehensive data specifically addressing Romanian women’s screening behaviours and the barriers they encounter in accessing or engaging in CCS.

To date, most studies examining CCS behaviours in Romania have taken a predominantly epidemiological or system-level perspective, with limited exploration of the subjective psychological and emotional dimensions that influence women’s decisions. Therefore, it is of utmost importance to address this research gap, and the present study directly responds to these needs by examining the emotional, cognitive, and social factors influencing Romanian women’s intention to undergo CCS, as well as the reasons behind their decisions to accept or decline screening.

## 2. Materials and Methods

This analysis utilized an online cross-sectional survey targeting Romanian women aged 18 and older to assess emotional, cognitive, and social factors associated with women’s intention to undergo CCS. The study received approval from the Ethical Committee of the Institute of Oncology “Prof. Dr. Ion Chiricuță”, Cluj-Napoca; reference number 254; date 19 January 2023.

### 2.1. Data Collection

The survey was distributed online in the form of a Google document, collecting responses between January 2023 and January 2025. The initial document was distributed by the investigators in the form of an invitation to access the document on various online social media platforms (Facebook, Instagram) and messaging apps (WhatsApp groups). To increase reach across various age and demographic groups. Potential participants were also invited to further distribute the invitation themselves and to their acquaintances, in a snowball sampling manner. Participation was anonymous and voluntary, and informed consent was obtained digitally prior to accessing the questionnaire, as a mandatory prerequisite together with the inclusion criteria.

### 2.2. Instruments

The questionnaire was developed based on existing literature. It included both closed-ended and open-ended questions to explore emotional reactions (e.g., shame, fear), cognitive beliefs (e.g., perceived risk, knowledge of HPV), social influences (e.g., peer or family support), and structural barriers (e.g., cost, accessibility) to CCS. It included 87 questions, aimed to explore cognitive, emotional, and social factors that could be associated with the intention to undergo CCS, based on previous published findings [33,34,35,36,37].

The first section of the questionnaire included the informed consent form, which offered information about the study’s aim. Demographic (5 questions) and medical history (7 questions) data (previous gynecology visits, diagnosis, and history of CCS) were also collected. Knowledge about HPV infection, CC, CCS, and the HPV vaccine (18 questions) was assessed by using both open-ended and closed-ended questions (dichotomic answers—yes/no, and multiple choice). After this, a short informational material regarding CCS’s purpose and specific procedures was offered. Open-ended questions were afterwards deployed to explore further reasons for accepting or declining CCS. The second part of the questionnaire included cognitive, emotional, and social factors assessments. Participants were asked to think about CCS (regarding CCS) and given 17 questions about the HPV infection/cervical cell changes (when I think about the HPV infection, including 5 questions), anticipating a diagnosis of HPV infection/cervical cell changes (If I would be diagnosed with the HPV infection/cervical cell changes, including 12 questions) and anticipating a diagnosis of CC (If I would be diagnosed with CC, including 20 questions). This section consisted of binary (yes/no) close-ended questions addressing cognitive appraisals, emotional reactions, perceived social influences, as well as the intention to undergo CCS (i.e., Do you want to get screened for cervical cancer?). The questionnaire can be found in Supplementary Materials Table S1.

### 2.3. Data Analyses

#### 2.3.1. Quantitative Analysis

Data analysis was performed using JASP Statistics 0.19.3 [38], an R statistics 4.5.1 software interface. Odds ratio (OR) was deployed to ascertain the association between the dichotomous variables, with a Confidence Interval (CI) of 95% [39] and a significant *p*-value of 0.05. Bivariate analysis was utilized to highlight the association between variables and intention to undergo cervical cancer screening. Fisher’s exact test was used when theoretically expected frequencies were less than 5 in >20% of the cases [40,41,42]. OR was used as an indicator for the power analysis to highlight the effect size of the association between variables. The following OR values for 1 degree of freedom (df) underscore the effect size: 1.5 to 1 represents a small effect, 2.5 to 1 = medium, 4 to 1 = large, and 10 to 1 = very large [43]. An OR = 0–0.4 indicates a large effect, an OR = 0.4–0.7 indicates a moderate effect, and an OR = 0.7–0.9 indicates a weak effect. Furthermore, the data was interpreted and analyzed, considering the power effect.

Descriptive statistics were used to explore demographic data, knowledge related to HPV, and medical history. The first section of the data analysis, descriptive statistics, covers demographic characteristics and personal history of gynaecological examinations and cervical cancer screening. The second part consists of different sections of the questionnaire in relation to cervical cancer screening intention: (1) Medical history, HPV knowledge; (2) Cervical cancer screening, the HPV infection; (3) Anticipating an HPV infection/cervical cell changes; (4) Anticipating a diagnosis of cervical cancer. The last three are distinguished based on the factual and anticipated nature of the questions, considering the cognitive, emotional, and social aspects associated with each section. The third section includes the qualitative analysis.

#### 2.3.2. Qualitative Analysis

The questionnaire contained two open-ended questions aimed at complementing the information gathered with open-ended questions, pertaining to (a) factors that prevented CCS, and (b) factors that prompted CCS.

Completed, open-ended responses were analyzed using an inductive content analysis approach [44]. The responses were exported in an Excel document and analysed manually. No software was used. Each response was coded by two independent researchers (N.-M.P. and D.T.); inter-coder discrepancies were solved by a third researcher (A.-S.B.). Responses were screened for keywords and common phrases in Romanian, used by participants, reflecting various reported barriers and facilitators, to derive the codes. Coding was performed using Romanian keyword-based matching, allowing responses to be assigned to one or more relevant themes. For example, a statement such as “*I don’t have money to do the test*” was coded under financial constraints, while “*Fear of the diagnosis*” was classified under emotional barriers. This approach ensured consistency and reproducibility while capturing the most salient motivations and obstacles reported by participants.

## 3. Results

### 3.1. Descriptive Analyses

The sample of the study consisted of 317 women who completed the questionnaire, with a mean (M) age of 35.02 (SD = 11.33). Response rate could not be estimated due to the intrinsic limitations of the recruiting method: snowball sampling. Most women were married (n = 144) or in a relationship (n = 91). Regarding educational levels, most held a Master’s degree (n = 112) or a Bachelor’s degree (n = 97). (See Table 1).

Table 2 shows participants’ history of gynaecological examinations and CCS. Over one in five participants (21.45%) had never undergone CCS practices, and almost 1 in 10 (9.14%) had never consulted a gyneacologist. A substantial proportion reported prior diagnoses of cervical cell changes (29.96%) or high-risk HPV strains (27.76%). Despite these data, HPV vaccination uptake was low, with only 16.08% of respondents indicating they had been vaccinated. Most women were aware of the HPV infection (94.95%), CC (99.68%), CCS (97.79%), and the HPV vaccine (93.69%). More than 90% of women believed that the infection could cause CC, and that it is asymptomatic at the beginning, while 46.05% perceived that they had little information regarding CCS, and 62.77% about the HPV vaccine (HPV-related knowledge can be found in the Supplementary Materials—Table S1).

### 3.2. Factors Associated with CCS Intention

#### 3.2.1. Medical History, HPV Knowledge, and Screening Intentions

Several factors were significantly associated with Romanian women’s intention to undergo CCS. A prior history of screening was strongly associated with future screening behaviour (OR = 7.564, CI = 3.523–16.241, *p* < 0.001), as was being diagnosed with an HPV strain (OR = 30.176, CI = 1.828–498.049, *p* < 0.001). A previous cancer diagnosis also correlated positively with screening intention (Fisher’s exact test OR = 0.200, CI = 0.063–0.703, *p* = 0.006). Awareness of the HPV vaccine was linked with greater screening intention (Fisher’s exact test OR = 5.566, CI = 1.722–16.649, *p* = 0.002). Little information about CCS (OR = 0.282, CI = 0.127–0.629, *p* = 0.001) and the HPV vaccine (OR = 0.341, CI = 0.137–0.853, *p* = 0.021) was also associated with intention, suggesting that perceived gaps in knowledge may act as motivational triggers. In addition, specific knowledge, such as knowing that HPV infection can be asymptomatic (Fisher’s exact test OR = 2.919, CI = 0.883–8.411, *p* = 0.040), that it can lead to cervical cancer (Fisher’s exact test OR = 4.398, CI = 1.117–14.994, *p* = 0.017), and that three doses are required for complete vaccine protection (OR = 2.533, CI = 1.203–5.332, *p* = 0.018), was positively associated with screening intent. Contemplating the HPV vaccine’s adverse effects was inversely associated with women’s intention to screen (OR = 0.439, CI = 0.212–0.909, and *p* = 0.034). The predominant concerns for adverse or secondary effects as stated by women were specific responses to any vaccine (n = 16), local reactions like pain (n = 14), fever (n = 9), potential infertility (n = 7), allergic reactions (n = 6), tiredness (n = 5), fainting (n = 3), muscular aches/weakness joint pain (n = 4), nausea (n = 4), vomiting (n = 3), headaches (n = 3), enlarged lymph nodes (n = 3), dizziness (n = 2) and chills (n = 2).

In contrast, several variables showed no significant association with screening intention. A previous diagnosis of cervical cell changes was not associated with increased screening behaviour (OR = 2.054, CI = 0.819–5.151, *p* = 0.159). General awareness of HPV infection (Fisher’s exact test OR = 1.243, CI = 0.131–5.822 *p* = 0.677), cervical cancer (Fisher’s exact test OR = 0, CI = 0–333.891, *p* = 1), and CCS (Fisher’s exact test OR = 3.577, CI = 0.328–23.001, *p* = 0.158) were not associated with intention to engage in screening. Likewise, vaccination status was not associated with screening intention (OR = 1.082, CI = 0.397–2.950, *p* = 1).

The power analysis of medical history and HPV-related knowledge yielded a very large effect size for several factors: being diagnosed with an HPV strain (OR = 30.176), a prior history of screening (OR = 7.564), awareness of the HPV vaccine (OR = 5.612), and knowledge that HPV can cause CC (OR = 4.432). Large effects were observed in completing the vaccine series (OR = 2.533), knowledge that the infection can be asymptomatic (OR = 2.919), a previous cancer diagnosis (OR = 0.200), and low information regarding CCS (OR = 0.282) and the HPV vaccine (OR = 0.341). Only knowledge regarding the vaccine’s adverse effects showed a moderate power effect (OR = 0.439)

#### 3.2.2. Cervical Cancer Screening, the HPV Infection, and Screening Intention

Several cognitive factors were found to be significantly associated with women’s intention to undergo cervical cancer screening. A strong positive association was observed between intention and believing in the benefits of screening (OR = 7.634, CI = 3.419–17.043, *p* < 0.001), and with perceived high susceptibility to HPV infection (OR = 3.380, CI = 1.551–7.368, *p* = 0.002). Conversely, a negative association emerged between screening intention and perceived personal efficacy in avoiding infection (OR = 3.643, CI = 1.687–7.868, *p* = 0.003), suggesting that confidence in one’s ability to avoid HPV may reduce motivation to engage in screening.

Intention was also negatively associated with difficulties in decision-making. Women who reported indecisiveness regarding screening procedures (OR = 0.261, CI = 0.124–0.548, *p* < 0.001) and difficulty making health-related decisions (OR = 0.296, CI = 0.136–0.645, *p* = 0.002) were less likely to express intent to screen. Additionally, the belief that screening is unnecessary in the absence of symptoms (Fisher’s exact test OR = 0.161, CI = 0.064–0.419, *p* < 0.001) was also negatively associated with screening intention.

Several cognitive factors were not associated with CCS intention. The belief that non-sexually active individuals do not require screening (Fisher’s exact test OR = 0.412, CI = 0.163–1.146, *p* = 0.053), having the same sexual partner (OR = 1.957, CI = 0.937–4.087, *p* = 0.096) and using protection (OR = 0.622, CI = 0.302–1.281, *p* = 0.262) results in low perceived susceptibility to being infected and were not associated with screening intention. This suggests that while those misconceptions exist among participants, they were not strong influencing factors in their stated screening intentions. The belief that gynaecological examination is painful (Fisher’s exact test OR = 0.566, CI = 0.192–2.036, *p* = 0.346), and the low perceived severity of the infection (Fisher’s exact test OR = 1.592, CI = 0.547–4.094, *p* = 0.307) was not associated with screening intention, nor were other health concerns (OR = 0.64, CI = 0.307–1.333, *p* = 0.245) and worries (OR = 0.563, CI = 0.272–1.167, and *p* = 0.14).

Emotional factors associated with women’s hesitation were the fear of having lesions that could potentially develop into cancer (OR = 2.385, CI = 1.148–4.956 and *p* = 0.023), the fear of receiving a cancer diagnosis (OR = 2.727, CI = 1.291–5.764 and *p* = 0.009) and feeling discouraged by the need to screen repeatedly (OR = 0.402, CI = 0.186–0.867, *p* = 0.023). These considerations promoted women’s reluctance to pursue screening. Other emotions associated with CCS intention comprised shame (OR = 0.461, CI = 0.223–0.956 and *p* = 0.048) and concerns about privacy violations (OR = 0.386, CI = 0.181–0.823 and *p* = 0.024). Additionally, fear about going to a gynaecologist (OR = 0.461, CI = 0.220–0.966 and *p* = 0.06), discomfort (OR = 0.579, CI = 0.279–1.201 and *p* = 0.145) and pain when considering undergoing a gynaecological examination (Fisher’s exact test = 0.566, CI = 0.192–2.036, *p* = 0.346) were not associated with the intention to participate in screening.

Social factors like support or encouragement from others (OR = 0.417, CI = 0.190–0.916, *p* = 0.034) a nd a doctor’s recommendation to screen (OR = 15.357, CI = 4.577–51.523, *p* < 0.001) were associated with screening intention.

The power analysis related to CCS and HPV infection showed a very large effect size for the doctor’s recommendation to screen (OR = 15.357) and understanding of the benefits of screening (OR = 7.634). Large effects were also observed in the perceived efficacy of the vaccine in protecting against HPV infection (OR = 3.643), high perceived susceptibility to contracting the HPV infection (OR = 3.380), the lack of symptoms (OR = 0.161), indecisiveness regarding CCS (OR = 0.261) and difficulty making health-related decisions (OR = 0.296) and privacy concerns (OR = 0.386). A moderate effect size was noted for fear of having lesions that could potentially develop into cancer (OR = 2.385), fear of receiving a cancer diagnosis (OR = 2.727), feeling discouraged by the need to screen repeatedly (OR = 0.402), feelings of shame (OR = 0.461) and support or encouragement received from others (OR = 0.417).

#### 3.2.3. Anticipating an HPV Infection/Cervical Cell Changes and Intention to Screen

When anticipating an HPV infection/cervical cell change, the prospective character of the variables showed a cognitive component even when considering emotions.

In anticipating an HPV infection only, the partner’s support positively correlated with women’s willingness to screen (OR = 6.016, CI = 2.808–12.888, and *p* < 0.001).

No cognitive factors, anticipated emotion, or sexual or relationship concerns were found to be associated with screening intention. Cognitive factors such as believing that being infected is terrible (OR = 0.487, CI = 0.224–1.06, *p* = 0.095), being less feminine (OR = 0.966, CI = 0.441–2.118, *p* = 1), and worrying about what might happen (Fisher’s exact test OR = 1.529, CI = 0.483–4.136, *p* = 0.413) were not correlated with the intention to screen. Anticipated emotions when thinking about the possibility of being infected or having cervical cell changes, guilt (OR = 0.561, CI = 0.258–1.222, *p* = 0.192), shame (OR = 0.659, CI = 0.320–1.358, *p* = 0.269) and fear of being diagnosed with cancer (OR = 1.234, CI = 0.561–2.714, *p* = 0.677) did not seem to be correlated with the intention to pursue screening. Also, there was no sense of guilt about potentially transmitting the virus to a partner that could be correlated with the intention to screen (OR = 1.789, CI = 0.867–3.695, *p* = 0.136). No sexual and relationship concerns or changes in sexual behaviours were associated with willingness to pursue screening, like decreased desire to have sexual intercourse (OR = 0.688, CI = 0.315–1.5, *p* = 0.449), low confidence (OR = 0.851, CI = 0.39–1.861, *p* = 0.846) and lower intimacy regarding sexual activity (OR = 2.067, CI = 0.928–4.605, *p* = 0.093), as well as the belief that partners would not want to have sexual intercourse (OR = 1.309, CI = 0.518–3.309, *p* = 0.663).

When anticipating an HPV infection or cervical cell changes, power effect analysis showed a large effect size regarding partners’ support (OR = 6.016).

#### 3.2.4. Anticipating a Diagnosis of Cervical Cancer and Cervical Cancer Screening Intention

When a diagnosis of CC is anticipated, two cognitive factors are associated with screening intention. Perceiving high severity of the CC, as shown by Fisher’s exact test = 5.464, CI = 1.535–17.701, and *p* = 0.004, and believing CC is treatable (Fisher’s exact test OR = 7.845, CI = 2.498–23.791, *p* < 0.001), were correlated with the intention to screen. Believing things would have gone differently if making another decision (OR = 1.358, CI = 0.557–3.31, *p* = 0.47) and worrying about what might happen (Fisher’s exact test, OR = 1.393, CI = 0.25–5.157, *p* = 0.489) were not linked to CCS’s intention.

Anticipated emotions correlated with screening intention were guilt of being diagnosed with CC (OR = 2.557, CI = 1.143–5.809, and *p* = 0.041) and fear of dying (OR = 2.253, CI = 1.073–4.727, and *p* = 0.037). At the same time, regret (Fisher’s exact test OR = 1.877, CI = 0.586–5.158, *p* = 0.24), shame (OR = 0.76, CI = 0.358–1.614, *p* = 0.551) and responsibility (OR = 1.151, CI = 0.535–2.478, *p* = 0.695) about being diagnosed with CC were not associated with the willingness to participate.

Regarding sexual and relationship concerns, no factors were correlated with screening intention. Sexual concerns like lack of desire for sexual intercourse (OR = 1.207, CI = 0.585–2.492, *p* = 0.709), low confidence regarding sexual activity (OR = 1.448, CI = 0.689–3.041, *p* = 0.33) and believing sexual contact could be painful (OR = 1.156, CI = 0.562–2.379, *p* = 0.716) were not associated with willingness to screen. Concerns about fertility or the possibility of having children after a diagnosis of CC did not seem to be correlated to the intention to undertake screening (OR = 1.212, CI = 0.585–2.512, *p* = 0.713). Regarding relationship concerns, perceiving communication as more difficult (OR = 0.551, CI = 0.258–1.178, and *p* = 0.139) and a lack of desire from the partner’s side to be intimate (Fisher’s exact test OR = 0.999, CI = 0.353–3.498, *p* = 1) were not associated with screening intention.

Regarding social factors, perceived partner support was associated with the intention to undergo screening (OR = 4.794, CI = 2.267–10.139, *p* < 0.001). While believing the partner will leave because of fertility concerns, not having children was not associated with screening intention (Fisher’s exact test OR = 0.921, CI = 0.295–3.837, *p* = 0.778)

When anticipating a diagnosis of CC, the power effect analysis revealed large effect sizes in the belief that CC is treatable (OR = 7.845), serious disease (OR = 5.464), and partners’ support (OR = 4.794). A large effect size appears regarding feelings of guilt if not undergoing screening (OR = 2.557). A medium effect size was revealed in fear of dying (OR = 2.253).

### 3.3. Qualitative Analysis of Barriers and Facilitators to CCS

**Figure 1:**
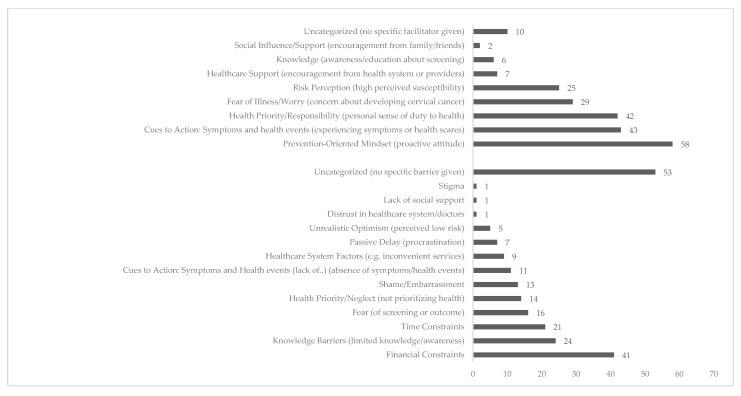
Barriers and Facilitators of Cervical Cancer Screening (numbers represent unstandardized frequencies).

To complement the quantitative findings, we coded and grouped participants’ open-ended responses into thematic categories reflecting the most salient barriers and facilitators to cervical cancer screening (CCS). Figure 1 presents the distribution of these themes, each representing a distinct motivational or obstructive factor mentioned by respondents.

Codes were not mutually exclusive; a single response could reflect multiple themes, allowing for a more nuanced representation of participants’ perspectives. Financial constraints, limited knowledge, time pressures, and fear emerged as dominant barriers. The verbatims, codes, and themes for factors that could act as barriers to screening intention can be found in Table 3. Conversely, the most frequently cited facilitators included a prevention-oriented mindset, personal health responsibility, and direct cues to action, such as symptoms or prior abnormal screening results. Emotional, informational, and structural influences were all represented among the responses, while some categories—such as social support or healthcare distrust—were rarely mentioned. The verbatims, codes, and themes for factors that could act as facilitators to screening intention can be found in Table 4.

Among barriers, the most frequently cited theme was **financial constraints** (n = 41), highlighting how direct costs associated with Pap smears, HPV testing, or related consultations represent a major obstacle, particularly in the absence of consistent public funding or accessible free services. This structural limitation was closely followed by **limited knowledge or awareness** (n = 24), with many women indicating that they lacked adequate information about screening procedures, the purpose of CCS, or the implications of HPV infection. These knowledge gaps reflect insufficient public education efforts and inconsistencies in health communication.

**Time constraint** (n = 21) was another common barrier, particularly among women balancing work, caregiving, and household duties. Emotional and psychological barriers also emerged prominently. **Fear of the procedure or its outcome** (n = 16), such as fear of a potential cancer diagnosis or worry about receiving bad news, deterred some women from engaging in screening, while others cited **shame or embarrassment** (n = 13) associated with undergoing a gynecological exam as a barrier. A distinct theme—**passive delay** (n = 7)—captured a subset of responses where women attributed their non-participation to procrastination, inertia, or lack of internal motivation rather than active avoidance. This theme was conceptually close to **health neglect or low prioritization** (n = 14), where screening was postponed simply because it was not considered as urgent or important as other life demands.

A smaller number of women referred to **healthcare system challenges** (n = 9), such as difficulty securing appointments, difficulty navigating within the healthcare system, dissatisfaction with past experiences, or perceived lack of support from providers. **Unrealistic optimism** (n = 4), or the belief that screening is unnecessary due to perceived good health or low risk, was also observed. Importantly, a significant portion of respondents (n = 53) were classified as uncategorized, often because they explicitly stated that they experienced no barriers or that they regularly attended screenings.

On the other hand, women considered that among the facilitators of screening was a proactive attitude toward health and a heightened awareness of personal vulnerability. The most prevalent theme was a **prevention-oriented mindset** (n = 58), reflected in statements about the importance of early detection, taking preventive action, or maintaining regular health checks. Intricately linked to this was the theme of **health responsibility and prioritization** (n = 42), where women expressed a strong internal drive to take care of their bodies and avoid future health complications. **Cues to action** (n = 43), such as previous abnormal results (including HPV strains or cervical cell changes), symptoms, or family history, played a significant role in motivating women to participate in CCS. These individual experiences increased awareness and urgency, encouraging women to pursue screening as a preventive measure.

**Fear of illness**, particularly fear of developing cervical cancer, was also a notable facilitator of screening (n = 29), with participants reporting that this anxiety pushed them toward action rather than avoidance. Relatedly, **risk perception** (n = 25) emerged as an influential motivator: women who perceived themselves as vulnerable to HPV infection or cervical disease were more likely to engage in screening behaviour. Less frequent but still meaningful themes included **support from the healthcare system or providers** (n = 7), **knowledge and understanding of CCS** (n = 6), and **social influence or encouragement from family or peers** (n = 2). We marked ten responses as **uncategorized** due to vagueness or ambiguity.

Taken together, the qualitative data emphasize that both emotional and cognitive factors play a crucial role in shaping CCS behaviour, alongside structural and contextual influences. While informational and systemic barriers continue to affect access, it is the interplay of personal beliefs, motivational readiness, and subjective risk awareness that most often drives screening uptake.

## 4. Discussion

The present study highlighted factors associated with Romanian women’s intention to undergo CCS and explored several reasons for engaging in CCS, both quantitatively and qualitatively. The quantitative analysis showed that a personal history of CCS, HPV infection, or cancer was associated with willingness to pursue CCS, in line with previous findings [45]. The qualitative analysis supports the findings, showing that previous abnormal CCS results (whether abnormal cytology or HPV infection) and family history are facilitating factors in the intention to undergo screening procedures. These findings are similar to published results on other populations [45,46]. Results highlight the important role of previous engagements with the healthcare system, whether through personal or family history. This also uncovers other issues regarding a significant percentage of the Romanian population who have never been screened. Moreover, in the study population, almost a quarter of respondents have never been to a gynecologist. Therefore, findings suggest the need for alternative strategies to reach out to those populations. A previous history of screening and a negative result could help women overcome their fear and adhere to the recommendation of preventive measures [46]. This highlights the importance of positive experiences and that once a participant is convinced to undergo screening, they will likely undergo it again. Reframing the screening process not as a negative tool raising the threat of a diagnosis, but as a preventive one, helped facilitate preventive measures and promoted earlier detection. This interplay depicts a certain typology of women willing to pursue screening, such as responsible subgroups that regularly undergo screening or willingly test themselves for HPV as part of opportunistic screening on the one hand, and a subgroup of participants driven out of fear on the other hand.

The present analysis was conducted on a highly educated population. This selection bias potentially skews results, uncovering that awareness of HPV infection, CC, and CCS was not associated with women’s intention to pursue screening; this lack of association may stem from the sampled population, where knowledge and awareness are no longer intrinsic limitations or vulnerabilities. In contrast, numerous other studies have found a significant correlation between increased awareness and intention to undergo screening [32,47]. The qualitative analysis, on the other hand, although different from the quantitative statistics, uncovered that a lack of awareness regarding CCS and CC could deter women from undergoing screening practices, potentially still highlighting the importance of awareness. Therefore, information campaigns can play a significant role in enhancing awareness among the rightfully selected populations, although they may not be sufficient for some women.

In the present study’s population, screening intention did not correlate with awareness; instead, it correlated with a deeper understanding and better knowledge about CC. Key knowledge factors, such as understanding that immunization requires three doses for protection, that HPV infection can be asymptomatic, and that it can lead to CC, were associated with women’s intention to be screened. This suggests that highly educated participants, understanding the intricate ways of developing and potentially preventing cancer, have higher chances of undergoing screening. Further strengthening results, the qualitative analysis uncovered that limited knowledge about the purpose and procedures of CCS, as well as the HPV infection, deterred women from screening; knowledge associated with the asymptomatic nature of the HPV infection could prompt women to pursue CCS. These findings suggest that specific personal experiences and detailed knowledge may matter more than general awareness or past preventive actions, such as vaccination.

Symptoms dually influenced participants’ preventive practices. When symptoms appeared, they often prompted individuals to seek screening, signalling that something may be awry with their health [48]. Qualitative findings highlighted that, besides symptom-driven behaviors, a prevention-oriented approach was valued by most women, positioning health as a personal responsibility, offering security for themselves but also for others, which could be reflected, firstly, by self-care practices highlighting an internal motivational component. Conversely, according to the quantitative analysis, knowing that CC could appear without symptoms in the early stages was associated with screening intention, possibly motivating women to engage in regular screenings and health check-ups even without specific warning signs. This intricate relationship between recognizing symptoms and understanding asymptomatic conditions underscores the need for vigilance and proactive health measures. Some women were only prompted to seek screening by the presence of symptoms. These results also strengthen the need for educational programs. Although highly knowledgeable, the women respondents in the present analysis were less aware that screening procedures are addressed to healthy asymptomatic individuals, and that symptoms should prompt diagnostic tests, rather than screening procedures. In both the quantitative and qualitative sections, the most common emotion that arose when participants reflected on engaging in CCS was the fear of a possible cancer diagnosis. As mentioned in previous studies, fear of a positive result, or anticipating a diagnosis of CC, discouraged women from undergoing screening, while possibly fostering a fatalistic attitude regarding a cancer diagnosis [32,49,50,51]. This further strengthens the need for educational programs that ensure appropriate knowledge is delivered correctly. While screening procedures are highly unlikely to fully diagnose a malignant condition, they have the role of selecting individuals who should undergo diagnostic procedures.

Perceiving a high susceptibility to infection and believing oneself is at-risk prompted women to screen. Perceived personal efficacy in avoiding the infection and lack of symptoms were inversely correlated with preventive behaviors. The qualitative analysis complemented this finding, showing that unrealistic optimism could also represent a barrier to screening intention. Consequently, passive delay due to low motivation or procrastination practices, health neglect, or low prioritization of preventive methods deters women from screening procedures. This further supports the need for proper information campaigns and risk awareness among even highly educated women.

As previously discovered, social support played a vital role in the intention to undergo screening, probably due to the complexities inherent in making health-related decisions [45,49,52]. Social and partner supports were associated with a complex decision-making process that ultimately influenced whether women engaged in screening, as highlighted by the results. Healthcare support, social influence, or encouragement from family or others was also mentioned as a facilitating factor in the qualitative analysis. Interestingly, Andreassen et al.’s study showed that those who were able to decide whether to attend screening had a higher attendance rate [47], while most women in this study showed a need for social support. Shame and embarrassment, pertaining especially to the gynecological examination, seemed to hinder women from screening despite their awareness of the importance of preventive measures [32,48,51,53,54,55,56]. The qualitative analysis enlightens a cultural component of shame, the cultural stigma associated with a lack of education on sexual behaviors or prophylaxis measures. The belief that gynecological examinations are painful was not associated with CCS intention; experiencing minimal discomfort encourages women to undergo regular check-ups [49,51,52], perhaps triggering anticipatory reactions to what a more serious diagnosis could provoke.

No anticipated emotions were associated with the intention to undergo cervical cancer screening when thinking about having an HPV infection or abnormal Pap results. This uncovers the lack of perceived severity attributed to infection. Anticipating a potential diagnosis of CC, combined with feelings of guilt stemming from a failure to undergo routine screening, played a pivotal role in the intention to pursue CCS in various other publications [57,58]. Conversely, regret was not correlated with the intention to undergo screening, as opposed to other findings that highlighted anticipated regret related to a diagnosis and its role as a motivational factor [25].

Although not the primary purpose of the present analysis, moving away from individual-level barriers, recommendations from HCP acted as a driver for women to pursue CCS. This was more nuanced in the qualitative analysis, highlighting trust in HCPs as pivotal in the decision-making process. This could be felt more acutely, especially in navigating the maze of a healthcare system without the guidance of an HCP. Results suggest the importance of a clearer pathway that patients could follow, potentially starting from education by HCP, receiving recommendations from primary care, and having experts guiding them through the process. It could also help mitigate the financial burden of the process, as some of the screening procedures are not completely reimbursed, and women might have to resort to opportunistic screening. In the open-ended questions, women stated that financial and time constraints were important factors that could act as barriers to their intention to screen. The financial constraints were attributed to competing priorities in the light of restricted financial resources and a lack of free access to screening, highlighting a need for free screening programs.

A variation in the effect sizes of different variables suggests potential practical implications of respective factors. For example, a larger effect size for variables such as a doctor’s recommendation and partner’s support than for factors such as fear and shame marks those characteristics as principal drivers for engaging in CCS, potentially providing valuable insights for future screening campaigns and initiatives.

### 4.1. Limitations

The population study comprised a group of participants who agreed to complete the questionnaire and had access to social media platforms or were acquaintances of respondents who had. Respondents had predominantly higher levels of education, were actively employed, and had a previous history of gynaecological examinations. As a result, the present findings might be less representative of those with a lower education level, unemployed or low socio-economic status, or who have never undergone gynaecological examinations and, implicitly, CCS. Although this might limit the generalizability of the results and represents a significant limitation of the analysis, the method chosen for enrolling ensured engagement from active members of the community and provided valuable feedback from this subpopulation. Furthermore, the questionnaire was distributed online in specialized forums, where most participants were actively engaging in screening conversations or were actively looking for specialized information. This method inherently limited the pool of respondents to those women who possessed basic knowledge and those participants who were already interested in screening practices or had basic knowledge about CCS. Nevertheless, over 21% of participants were never screened, potentially revealing valuable information regarding this subpopulation and therefore lessening some limitations.

The study enrollment method utilized social media distribution and relied on snowball sampling to further recruit additional respondents. The snowball sampling strategy is a non-probabilistic method that relies on initial participants to enrol other women by further inviting them and distributing the questionnaire. This inherently has disadvantages, such as the prolonged enrollment timeframe. Results were obtained after an accrual period of almost 2 years. Moreover, this technique renders estimating the response rates impossible, since the investigators no longer had control over the invited populations, nor an accurate way to count the number of invited participants. These are significant biases of the present analysis that cannot be mitigated. However, this chain-referral invitation presents other advantages that were useful in the study scenario. It allowed investigators to reach out to other varied populations who initially had no access to the internet and social media platforms and would not have been reached otherwise. Therefore, almost a quarter of respondents have never been to a gynecologist. This aligns with current estimates suggesting that around 25% of Romanian women had never been screened during their lifetime [59]. This enriches results with varied populations and enhances the generalizability.

Regarding the questionnaire itself, a limitation is represented by the limited number of open-ended questions utilized: only two questions enable open answers. While this enriches findings by allowing some qualitative analyses to be deployed, it limits respondents’ answers and further in-depth statistics. This can lead to a saturation of themes. A larger population and more open-ended questions could have mitigated these and would have offered a more holistic overview. However, there is a high probability that more open answers would have deterred participants from enrolling or responding.

Due to their high educational levels and access to information, most women who completed the questionnaire were aware of HPV infection, CCS, CC, and the HPV vaccine. This potentially skews the results of this study, while other investigations highlighted lower levels of awareness and knowledge in the Romanian population. [60,61,62]. Regardless, valuable insight was gained from exploring this subgroup, which can further act as a pillar for developing strategies to mitigate the poor addressability of screening programs, even in these educated and knowledgeable groups. Moreover, the study population is limited to Romanian women. This was not arbitrary, but the country has recently adopted a National Plan for Combating Cancer. Recent endeavours strive to create an organized program and primary prophylaxis through immunization and secondary prevention, including HPV testing and Pap smears, which are important milestones to be covered. Therefore, such population-level exploratory analyses can serve as valuable insights.

### 4.2. Future Directions

Future research should explore techniques that enhance awareness and knowledge of HPV, including primary and secondary preventive measures available. Informative campaigns should strive to offer clearer pathways and include essential steps to follow a positive screening result. As expressed by many respondents and previous findings, individual barriers are only part of the issues deterring women from screening. It is crucial for policymakers to elaborate an organized screening program that proactively reaches out to women, making the screening process more accessible and navigable. By addressing financial barriers and providing clear guidance, such a program could significantly improve women’s health outcomes and empower them to take charge of their health. Moreover, invitation systems, organized registries, results delivery, and follow-up for both negative and positive results could ensure inclusive tactics reach out to all populations.

The emotional barriers faced by women revolve around anticipating an oncological diagnosis or undergoing gynecological examinations. These barriers are compounded by feelings of shame and fear, as well as a profound sense of vulnerability during gynecological examinations, where privacy can feel elusive. To effectively navigate these challenges, it is essential to implement targeted interventions that address and alleviate these deeply rooted emotions, fostering a more supportive and understanding environment for women during such vulnerable moments. As mentioned above, a strong correlation with very large effect sizes was uncovered when it comes to the doctor’s recommendation on engaging in CCS. It is imperative to mention the pivotal role of General Practitioners or Family Care Doctors in Romania, who, most of the time, are the first contact or encounter with the medical system for many vulnerable patients, especially in more isolated areas. As such, perhaps primary care medicine should prompt women to undergo screening, while a comprehensive analysis of the current relationship between primary care and screening should be undertaken to evaluate the current role of General Practitioners in screening practices. Some other strategies to mitigate feelings of vulnerability or shame could foster the role of self-sampling as an alternate method for screening. It has been previously uncovered that self-sampling could aid with overdue patients for screening appointments [63], and perhaps larger-scale implementation could enhance addressability.

Apart from strategies mitigating system-level barriers, individual factors should be taken into consideration. Support from others and partners should be explored further to highlight the essential topics that can act as cornerstones for decision-making while exploring the difficulties faced with health-related decisions. This exploration is especially vital when navigating the complex challenges of health-related choices and determining what aspects women prioritize in the decision-making process. Although it was not the purpose of the present investigation, enhancing knowledge, awareness, and education on CC and CCS and immunization was shown to mitigate the low addressability. Against a borderline individual-level barrier, enhancing knowledge through educational programs is a pivotal aspect that ensures an effective healthcare system. Future endeavours should explore the role of educational programs implemented as early as possible, perhaps through school-based programs, and assess subsequent results. As an intricate interplay between shame, guilt, emotions, and support from social groups, it is vital to readdress the roles of HCP as prompts for preventive care.

Moreover, the present analysis uncovered several determinants of screening engagement and preventive behaviors in more developed populations where knowledge and awareness are no longer an intrinsic barrier. Although some studies focused on underserved and vulnerable women, it is of paramount importance to address individual- and system-level barriers to screening in the general populations that do not face the same vulnerabilities.

## 5. Conclusions

This study identified emotional and cognitive factors associated with women’s intention to undergo CCS. Emotional reactions can include feelings of shame, concerns about privacy while undergoing gynecological examinations, fear of undergoing screening, and anticipating a possible cancer diagnosis. Lack or limited knowledge regarding HPV, along with a lack of awareness about the advantages of regular screening, can lead to a false sense of confidence in women’s ability to protect themselves from HPV infection. This interplay between emotions and beliefs created challenging barriers discouraging women from engaging in CCS. Lack or limited knowledge regarding HPV, along with a lack of awareness about the advantages of CCS, could deter women from screening. The financial burden and perceived low susceptibility to infection acted as barriers to the screening addressability. Findings serve as pillars for creating and perfecting organized screening programs in various populations, from vulnerable women who never screened to more knowledgeable populations where education and lack of information no longer represent intrinsic barriers.

## Figures and Tables

**Table 1:** Demographic characteristics of participants.

Variable		Number	Percentage
Civil status	Single	62	19.55%
	In a relationship	91	28.7%
	Married	144	45.42%
	Divorced	18	5.67%
	Widowed	2	0.63%
Education	Secondary school or less	10	3.15%
	Highschool	76	23.97%
	Postsecondary school	9	2.83%
	Bachelor’s degree	97	30.59%
	Master’s degree	112	35.33%
	Postgraduate degree	13	4.1%
Occupational status	Employed	229	72.23%
	Unemployed	18	5. 67%
	Student	61	19.24%
	Retired	9	2.83%
Income	Under 1000 RON	39	12.3%
	1000–2500 RON	63	19.87%
	2500–3500 RON	51	16.08%
	3500–4500 RON	56	17.66%
	Above 4500 RON	108	34.06%

**Table 2:** History of gynaecological examinations and cervical cancer screening.

Variable		Number	Percentage
When did you last undergo a gynaecological examination?	Less than a year ago	169	53.31%
	1–2 years ago	64	20.18%
	2–3 years ago	14	4.41%
	3–5 years ago	15	4.73%
	More than 5 years ago	26	8.20%
	I have never been to a gynaecologist	29	9.14%
Have you ever been screened for CC? (Pap smear/HPV test)	No	68	21.45%
	Yes	249	78.54%
How long ago did you get screened for CC (Pap smear/HPV test)?	Less than a year ago	144	45.42%
	1–2 years ago	55	17.35%
	2–3 years ago	18	5.67%
	3–5 years ago	12	3.78%
	More than 5 years ago	22	6.94%
	I have never been screened	66	20.82%
Have you been diagnosed with cervical cell changes?	No	222	70.03%
	Yes	95	29.96%
Have you been diagnosed with certain HPV strains?	No	229	72.24%
	Yes	88	27.76%
Have you been diagnosed with any cancer?	No	299	94.32%
	Yes	18	5.67%
Are you vaccinated against HPV?	No	266	83.91%
	Yes	51	16.08%

**Table 3:** Verbatims, codes, and themes—cervical cancer screening barriers.

Barriers		
Verbatim	Codes	Themes
“Lack of symptoms, and lack of money.”	Financial limitations	Financial constraints
“The budget is already limited; I can’t afford a test.”	Competing financial priorities	
“I kept postponing it, but also the fact that it is not a procedure covered by health insurance.”	Lack of free screening	
“I don’t know what an HPV test is about.”	Low awareness	Limited knowledge or awareness
“I don’t need to screen, if I don’t have any symptoms.”	Lack of necessity for screening	
“The work schedule that needs to be changed in order to visit the gynecologist.”	Scheduling conflicts	Time constraint
“I have 0 free time.”	Lack of time	
“Poor time management.”	Inefficient time prioritizations	
“The gynaecological examination is not the most comfortable exam.”	Discomfort with procedure	Fear of the procedure or its outcome
“Fear of a cancer diagnosis.”	Fear of results	
“The only thing that deters me from undergoing cervical cancer screening it’s convenience and the simple—embarrassment of going to a gynaecologist.”	Embarrassment	Shame or embarrassment
“The culture of shame, lack of education on this topic and the prophylaxis.”	Cultural stigma	
“Constantly postponing.”	Procrastination	Passive delay
“In my opinion, there are two major causes: laziness (indifference) and lack of education.”	Low motivation	
“What is currently preventing me from undergoing screening is my focus on other concerns.”	Competing priorities	Health neglect or low prioritization
“I don’t think it’s necessary.”	Perceived lack of importance	
“Prioritization, difficulty in finding a doctor who is sufficiently empathetic and gentle (distrust of the human side of doctors).”	Lack of providers’ support	Healthcare system challenges
“The twisted medical path.”	Complex healthcare system navigation	
“It cannot happen to me”	Optimistic bias	Unrealistic optimism

**Table 4:** Verbatims, codes, and themes—cervical cancer screening facilitators.

Facilitators		
Verbatim	Codes	Themes
“The desire to be healthy and prevent, rather than treat.” “Prevention is more efficient than treatment.”	Emphasis on prevention	Prevention-oriented mindset
“Caring for my body.”	Self-care	Health responsibility and prioritization
“The need for and importance of knowing that I am okay.”	Health awareness	
“Responsibility, the desire to take care of oneself, the need for security, awareness of the importance of health and disease prevention, and more.”	Personal responsibility	
“A healthy life, a pregnancy without issues and avoiding the transmission of this virus to my partner.”	Protecting self and others	
“Knowing that I have the HPV infection”	Personal health history	Cues to action
“A family history of cervical cancer”	Family history	
“Symptoms in the genital area/lower abdomen!”	Symptoms prompt screening	
“Fear of disease, fear of suffering, hope that early detection can lead to treatment.”	Disease-related fear	Fear of illness
“Too many women are dying.” “Not to die from a 90% preventable cancer.”	Fear of dying	
“The risk of cancer and the possibility of its early detection.”	Perceived vulnerability	Risk perception
“Sexual intercourse with multiple partners.”	Behavioral risk factors	
“Age, medical education, gynecologist’s advice, and life experience.”	Healthcare professional recommendation	Support from the healthcare system or providers
“I trust the advice of doctors; I know that such a medical procedure makes a difference for many people.”	Trust in Healthcare practitioners	
“Information related to screening and the disease.”	Knowledge of CCS	Knowledge and understanding of CCS
“The fact that it is an asymptomatic, insidious disease.”	Awareness of the asymptomatic nature	
“In my family, my mother had cervical problems, and my grandmother had lung cancer.”	Family influence	Social influence or encouragement from family or peers

## Data Availability

Data available on request.

## Supplementary Materials

The following supporting information can be downloaded at: https://www.mdpi.com/article/10.3390/healthcare13172147/s1, Table S1. HPV related knowledge.

## Abbreviations

The following abbreviations are used in this manuscript:

HPV	Human Papilloma Virus
CCS	Cervical Cancer Screening
HBM	Health Belief Model
TPB	Theory of Planned Behaviour
CC	Cervical Cancer
OR	Odds Ratio
CI	Confidence Interval
M	Mean
SD	Standard Deviation
