# Supplementary material for: Emotional, Cognitive, and Social Factors Influencing Romanian Women’s Intention to Undergo Cervical Cancer Screening: A Mixed-Method Study
Source: Healthcare (Basel). 2025 Aug 28;13(17):2147. doi: 10.3390/healthcare13172147 (PMC12427809; doi:10.3390/healthcare13172147)
Supplement: Supplementary file 1 [file healthcare-13-02147-s001.zip › healthcare-3798611-supplementary.pdf]

Table S1. HPV related knowledge

Variable		Number	Percentage
**Knowledge about the HPV infection**			
Have you heard of Human Papilloma Virus (HPV) infection?	No<br>Yes	16<br>301	5.04 %<br>94.95 %
Do you think this infection can cause cervical cancer?	No<br>Yes	16<br>301	5.04 %<br>94.95 %
Do you think the infection is asymptomatic at first?	No<br>Yes	26<br>291	8.2 %<br>91.79 %
Can only people who have more than one sexual partner be infected?	No<br>Yes	281<br>36	88.64 %<br>11.35 %
What other cancers can it cause? (you can choose more than one answer)	Cervical Cancer<br>Oropharynx<br>Skin<br>Vagina<br>Penis	292<br>148<br>47<br>186<br>134	92.11 %<br>46.68 %<br>14.82 %<br>58.67 %<br>42.27 %
How can the infection be transmitted? (you can choose more than one answer)	By touch<br>Through blood<br>Through sexual intercourse<br>Through saliva<br>Through air	78<br>101<br>302<br>41<br>1	24.60 %<br>31.86 %<br>95.26 %<br>12.93 %<br>0.32 %
**Knowledge about Cervical Cancer**			
Have you heard of cervical cancer?	No<br>Yes	1<br>316	0.31 %<br>99.68 %
Can vaccination prevent some types of cervical cancer?	No<br>Yes	29<br>288	9.14 %<br>90.85 %

Can it be detected in the early stages by screening? (Pap Test/ HPV Test)	No<br>Yes	2<br>315	0.63 %<br>99.36 %
Can cervical cancer be treated and cured if found early?	No<br>Yes	9<br>308	2.83 %<br>97.16 %
**Knowledge about cervical cancer screening and HPV vaccine**			
Have you heard about cervical cancer screening (Pap smear/HPV test)?	No<br>Yes	7<br>310	2.2 %<br>97.79 %
I have too little information about cervical cancer screening	No<br>Yes	171<br>146	53.94 %<br>46. 05%
Have you heard about the HPV vaccine?	No<br>Yes	20<br>297	6.3 %<br>93.69 %
I think the vaccine may have adverse/secondary effects	No<br>Yes	201<br>116	63.4 %<br>36.59 %
Cervical cancer screening should continue after vaccination	No<br>Yes	16<br>301	5.04 %<br>94.95 %
I have too little information about the vaccine	No<br>Yes	118<br>199	37.22 %<br>62.77 %
3 doses of vaccine are needed for full protection	No<br>Yes	78<br>239	24.6 %<br>75.39%
